# TIME-OF-DAY MOVEMENT AMOUNT AND VARIABILITY BY COGNITIVE STATUS IN A SAMPLE OF BLACK OLDER ADULTS

**DOI:** 10.1093/geroni/igae098.4094

**Published:** 2024-12-31

**Authors:** Amal Wanigatunga, Francesca Marino, Fangyu Liu, Nicola Shen, Marta Karas, Lauren Parker, Marilyn Albert, Jennifer Schrack

**Affiliations:** Johns Hopkins Bloomberg School of Public Health, Baltimore, Maryland, United States; Boston University School of Medicine, Boston, Massachusetts, United States; Johns Hopkins Bloomberg School of Public Health, Baltimore, Maryland, United States; Boston Public Health Commission, Boston, Massachusetts, United States; Takeda Pharmaceuticals, Boston, Massachusetts, United States; Johns Hopkins Bloomberg School of Public Health, Baltimore, Maryland, United States; Johns Hopkins University School of Medicine, Baltimore, Maryland, United States; Johns Hopkins Bloomberg School of Public Health, Baltimore, Maryland, United States

## Abstract

Reduced daily movement, particularly in the mornings, is linked with elevated Alzheimer’s disease risk, yet prior studies have consisted of primarily non-Hispanic White participants. Whether this association exists in Black older adults, who often are underrepresented in research and experience delays in cognitive impairment diagnosis, remains unexplored. Wrist-worn accelerometry was analyzed from 21 Black participants (median age: 73 years, 83% women) referred from the Johns Hopkins Alzheimer’s Disease Research Center who evaluate each participant for mild cognitive impairment (MCI). Separate age and sex adjusted linear regression models estimated movement differences by MCI status in mean: 1) total physical activity amount or variability (counts/d) over 24-hrs and 2) amount or variability of activity (counts/d) from 12am-6am, 6am-12pm, 12pm-6pm, and 6pm-12am. Variability measures were calculated as the SD of minute-level activity during each time period. Thirteen participants (62%) were identified with MCI. Participants with MCI averaged 1,700,927 (SD=721,021.9) counts/d compared to cognitively unimpaired participants who averaged 1,727,588 (SD=841,627.9) counts/d. Amount of activity counts/d did not differ between those living with and without MCI overall or by time-of-day (p for all>0.56). However, there was a significant difference in physical activity variability between 6am-12pm, where variability was higher among those with MCI by 66,451 (95% CI: 883, 132,020) counts/d. Our findings warrant further investigation into whether variability, rather than amount, in everyday movement occurring during early morning hours could serve as a digital biomarker to enhance cognitive impairment screening for Black older adults living independently.

